# Errata: I Diretriz Brasileira de hipertensão arterial na diálise da Sociedade Brasileira de Nefrologia

**DOI:** 10.1590/2175-8239-JBN-2024-0033er

**Published:** 2026-02-06

No artigo “I Diretriz Brasileira de hipertensão arterial na diálise da Sociedade Brasileira de Nefrologia”, com número de DOI: https://doi.org/10.1590/2175-8239-JBN-2024-0033pt, publicado no periódico Brazilian Journal of Nephrology, 47(1):e20240033, 2025, na página 12:

Onde se lia:

**Quadro 3** Protocolo para realização da monitorização ambulatorial da pressão arterial (MAPA) nos pacientes em diálise, segundo as *Diretrizes brasileiras de medidas da pressão arterial dentro e fora do consultório* – 2023^119^

**Monitorização Ambulatorial da Pressão Arterial (MAPA)**

- **Dia de instalação:** na HD deve ser instalada após a sessão de diálise do meio da semana e retirada imediatamente **após** a sessão seguinte.

Leia-se:

**Quadro 3** Protocolo para realização da monitorização ambulatorial da pressão arterial (MAPA) nos pacientes em diálise, segundo as *Diretrizes brasileiras de medidas da pressão arterial dentro e fora do consultório* – 2023^119^

**Monitorização Ambulatorial da Pressão Arterial (MAPA)**

- **Dia de instalação:** na HD deve ser instalada após a sessão de diálise do meio da semana e retirada imediatamente **antes** da sessão seguinte.

Prof. Dr. Miguel C. Riella

*Editor-chefe*

